# A Novel Low Right Atrial–approach Transseptal Puncture Technique for Large-bore Deflectable Sheaths: Enhancing Control, Precision, and Safety

**DOI:** 10.19102/icrm.2026.17031

**Published:** 2026-03-15

**Authors:** Esteban Martin Kloosterman

**Affiliations:** 1 Cardiac Arrhythmia Service, Boca Raton, FL, USA

**Keywords:** Left atrial appendage occlusion, large-bore sheath, left atrial access, pulsed field ablation, transseptal puncture

## Abstract

The conventional transseptal puncture (TSP) technique, developed in the 1960s, remains a procedural standard but offers limited control when applied to contemporary large-bore deflectable sheaths. The objective of this study is to describe a novel low right atrial (RA)–approach TSP technique designed to optimize control, precision, and safety when using large-bore deflectable systems. This technique eliminates the need for superior vena cava (SVC) positioning and instead advances the deflectable sheath–dilator assembly from the low RA toward the interatrial septum under imaging guidance. The deflectable capabilities of the sheath, protected by the spiral or J-tip wire, enable atraumatic manipulation and precise targeting before puncture. This method was used in 63 consecutive procedures, including pulsed field ablation, left atrial appendage occlusion, and concomitant cases. All punctures were completed successfully without complications. The average time from inferior vena cava advancement to septal crossing was 2–3 min. This new low RA–approach TSP technique provides superior sheath control and maneuvering of large-bore deflectable systems, offering competitive safety and efficiency as an excellent alternative to conventional SVC-based approaches.

## Introduction

**Figure 1:**
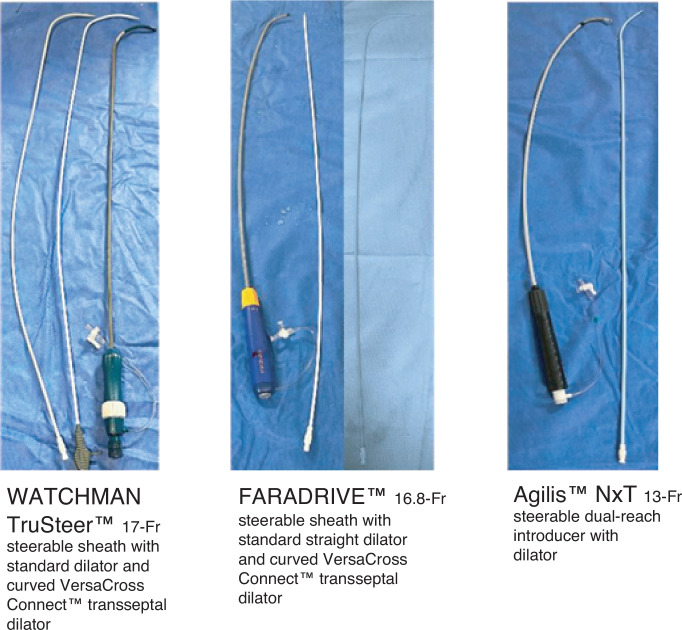
Three samples of large-bore sheaths and dilators used in these series. The Agilis system can use the spiral or J VersaCross wire, but not the VersaCross dilator, as such would compromise the sheath valve seal.

Since its inception in the 1960s, transseptal puncture (TSP) has been an essential technique for accessing the left atrium in interventional electrophysiology and structural heart procedures.^1–3^ The classic “pull and drop” approach—advancing a small sheath over a wire to the superior vena cava (SVC) and subsequently exchanging the wire for a fixed-curve needle, which is then pulled down until it drops into the interatrial septum (IAS)—has proven effective for decades. Over the past 10 years, a shapeable introducer with a blunt tip has allowed for improved and safer transseptal system manipulation, along with the subsequent introduction of radiofrequency (RF) needles and then wires to replace the needle.^4–6^ These advancements allowed for completion of a less-traumatic transseptal crossing due to requiring less tenting pressure. However, with the advent of complex atrial fibrillation (AF) ablations, left atrial appendage occlusion (LAAO), and transcatheter mitral interventions, larger and stiffer sheaths are now required **(Figure 1)**. These new devices pose challenges for the conventional method, including limited control of the puncture location, excessive septal tenting, and difficulty in repositioning once contact is made.

To avoid these situations, operators often resort to the use of a conventional transseptal system to be exchanged thereafter for a large-bore sheath over the wire. However, this increasing use of large-bore deflectable sheaths has encouraged the need for a more efficient transseptal procedure. The currently in use deflectable capabilities of the sheaths provide an opportunity to improve precision and reduce procedural risk. We propose a novel low right atrial (RA)–transseptal approach, leveraging sheath deflection ability using a protective spiral or J wire to achieve controlled, atraumatic septal engagement and puncture.

**Figure 2:**
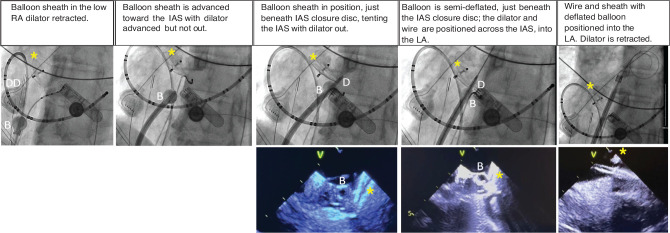
Fluoroscopic and intracardiac echocardiography sequence of balloon transseptal sheath (SafeCross™) used in a very precise interatrial septum (IAS) puncture just beneath an IAS occlusion double disc. The balloon sheath is advanced from the low right atrium up and directed to the IAS in the left anterior oblique view with clear confirmation on intracardiac echocardiography. *Abbreviations:* RA, right atrium; IAS, interatrial septum; LA, left atrium. *Notes:* B, balloon at the distal end of the deflectable transseptal sheath; D, dilator; DD, duadecapolar catheter along the crista terminalis and tricuspid valve isthmus, into the coronary sinus. *IAS double disc closure device.

The concept of starting the transseptal maneuver from the low-mid RA instead of the SVC was derived from our use of a different system. In 2022, a steerable balloon-tip sheath RF introducer system became available allowing sliding of the sheath tip over the IAS to reach very specific puncture location sites (SafeCross™ Transseptal RF Puncture; East End Medical I, LLC, Hollywood, FL, USA) **(Figure 2)**.

## Methods

### Procedural overview

**Figure 3:**
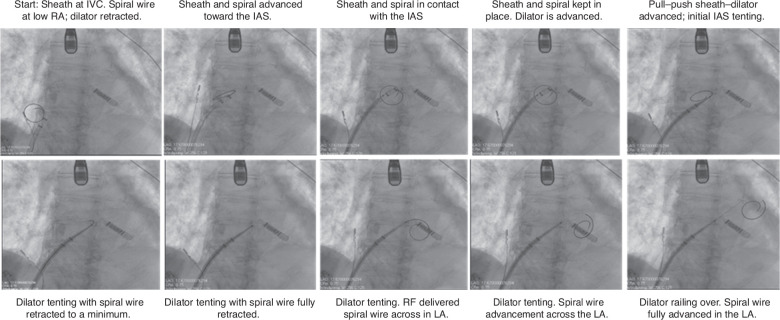
Example 1. Patient undergoing pulsed field ablation for atrial fibrillation. Fluoroscopic sequential series of sheath positions applying the described new technique. *Abbreviations:* IAS, interatrial septum; IVC, inferior vena cava; LA, left atrium; RA, right atrium.

**Figure 4:**
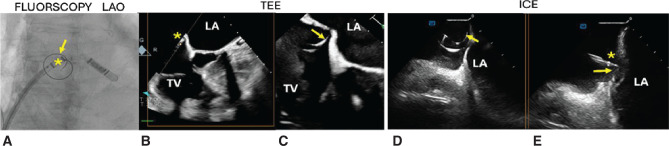
Fluoroscopic **(A)**, transesophageal echocardiography **(B, C)**, and intracardiac echocardiography **(D, E)** correlation of the sheath and wire in contact with the interatrial septum (IAS). *Tip of the sheath with slight tenting of the IAS. Arrows indicate the spiral wire in contact with the IAS. *Abbreviations:* LA, left atrium; LAO, left anterior oblique; ICE, intracardiac echocardiography; TEE, transesophageal echocardiography; TV, tricuspid valve.

**Figure 5:**
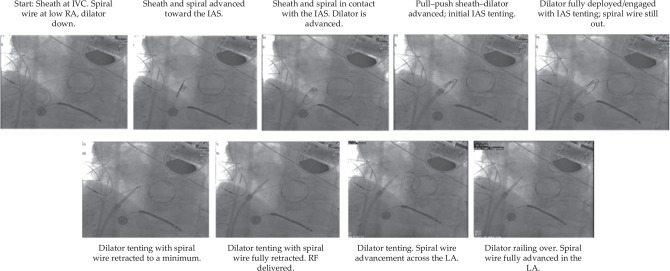
Example 2. Patient with DDD-mode implantable cardioverter-defibrillator, mitral valve replacement undergoing concomitant procedure. Fluoroscopic sequential series of sheath positions applying the new technique described. *Abbreviations:* IAS, interatrial septum; IVC, inferior vena cava; LA, left atrium; RA, right atrium; RF, radiofrequency.

1. After right femoral venous access is obtained, an RF-compatible spiral or J-tip guidewire is advanced to the mid-to-low RA. Separately, the deflectable sheath–dilator assembly is advanced to the RA–inferior vena cava (IVC) junction, with the dilator partially retracted inside the sheath (to allow full sheath deflection later). The sheath remains neutral (non-deflected) at this stage.
2. Next, under transesophageal echocardiographic (TEE), intracardiac echocardiographic (ICE), or fluoroscopic guidance, the sheath can be advanced and deflected as needed to the desired IAS region. The wire serves as a septal protector, allowing the sheath to slide into position without exerting pressure.
3. Of note, the large deflectable sheath mechanism is not meant to be used with the dilator engaged, hence the following maneuver:
  - Once the sheath and wire are on target against the septum, the dilator is advanced a few millimeters beyond the sheath tip to achieve minimal tenting.
  - Maintaining the position of the dilator, the sheath is gently pulled back to re-engage with the dilator to form a single unit. In a “pull–push” fashion, the dilator can be further pushed to increase tenting while the sheath is being pulled back.
  - Applying some more forward pressure to stabilize the system in the desired location, the wire is then brought back into the dilator. After confirming adequate position and tenting, RF energy (1–2 s) is delivered, advancing the wire to cross the IAS.
4. The sheath–dilator system is advanced over the wire into the left atrium as per standard technique **(Figures 3–5)**.

### Fluoroless application

The proposed technique can be performed without fluoroscopy using TEE and/or ICE and, if part of the procedure, using three-dimensional electroanatomic mapping.

To ensure accurate wire–sheath relationships, one should adopt the following steps:

- Pre-mark the wire positions relative to the sheath and dilator.
- Apply subtle bends for tactile feedback.
- Correlate wire deflection and impedance changes on mapping systems (caution note: the wire, when inside the sheath, not the dilator, may not achieve a significant impedance change to accurately reflect if it is in or out of the sheath).

ICE-guided procedures benefit from matching sheath handle orientation with the imaging probe to maintain consistent spatial reference.

This manuscript describes a technique applied during routine procedures using standard-of-care tools, all of which are encompassed within the standard general hospitalization and procedure consent of the patients involved. All presented data have no patient identifiers or any personal patient information and therefore were deemed exempt from a need for approval by an investigational review board (Human Studies Committee or Ethics Committee or Animal Care and Use Committee).

See **Table 1** for all hardware specifications and manufacturer information.

**Table 1:** Hardware Specs

FARADRIVE™: 16.8-Fr steerable sheath with a standard dilator (Boston Scientific) WATCHMAN TruSteer™ Access System: 17-Fr outer steerable sheath (Boston Scientific) Dilator: VersaCross Connect™ Access Solution with TRUform™ Shapeable Technology (Boston Scientific) Wire: VersaCross RF wire is available as a J-tip or pigtail (spiral) wire (Boston Scientific) WATCHMAN PRO (Boston Scientific) Agilis™ NxT Steerable Introducer, Dual-Reach, 13-French (Abbott, Chicago, IL, USA) SafeCross™ Transseptal System (East End Medical I LLC, Miramar, FL, USA)

## Results

The technique was applied in 63 consecutive patients undergoing large-bore TSP, including: 31 LAAO cases treated with WATCHMAN implantation (Boston Scientific, Marlborough, MA, USA) (all TEE-guided, with 7 also using ICE), 21 AF cases treated with pulsed field ablation (PFA) (all ICE-guided), and 11 concomitant PFA + WATCHMAN cases (TEE-/ICE-guided).

Successful left atrial access was achieved in all procedures with no complications. Also, the target puncture site was obtained on the first attempt in all cases—that is, the sheath never had to be withdrawn all the way back to the IVC. Instead, if the sheath was deflected toward the septum and found to be too anterior, posterior, low, or high, by simply relaxing the curve of the deflection, the sheath could then be maneuvered and redirected with the proper correction.

The average time from sheath advancement at the IVC to completed transseptal crossing was 2–3 min (including imaging documentation).

The VersaCross Connect™ RF system with the TRUform™ shapeable dilator was preferred with the FARADRIVE™ and TruSteer™ deflectable sheaths (all Boston Scientific), though standard dilators were also effective.

## Discussion

This novel low RA transseptal approach optimizes the inherent advantages of modern deflectable sheaths by maintaining full control of direction and pressure during positioning. By eliminating the need for SVC advancement and pull-drop down, the technique:

- Avoids the need for transseptal system exchanges.
- Enhances precision in targeting the fossa ovalis or alternative septal regions.
- Reduces excessive tenting, particularly in thin or compliant septum.
- Minimizes patent foramen ovale–related misdirected crossings.
- Avoids the eventual need for repeated drop-down attempts.
- Avoids lead entanglement in patients with cardiac implantable devices.
- Allows seamless execution in fluoroless workflows.

For less-experienced operators, the technique simplifies navigation and positioning and provides control in the execution, avoiding recurrent attempts at sheath positioning. For advanced users, it offers an option for enhanced efficiency and accuracy when using large-bore, heavy, or double-curved sheaths.

### Limitations

This experience reflects the outcomes from a single center and a single operator and thus requires further validation. The patient cohort consisted of consecutive, unselected cases, encompassing a wide range of procedures, the presence of device leads, and prior cardiac/valve surgeries. Despite this diversity, no specific limitations to the low RA transseptal approach were identified; in fact, the results from these varied scenarios favor its use.

## Conclusion

In these series, the low RA–approach TSP technique using large-bore deflectable sheaths provided improved control, safety, and procedural efficiency compared to the experience with the traditional SVC-based method. In 63 consecutive cases, it yielded 100% procedural success, with no complications. This method represents a refined approach for modern electrophysiology and structural heart interventions requiring large-bore transseptal access.
